# Overview on Epstein–Barr Virus–Host Interactions With a Focus on Host Cell Transcriptome Alterations

**DOI:** 10.1002/cbf.70322

**Published:** 2026-10-14

**Authors:** Louise Wallot, Tim Hohmann, Urszula Hohmann, Faramarz Dehghani, Olaf Grisk, Simon Jasinski‐Bergner

**Affiliations:** ^1^ Institute of Physiology, Brandenburg Medical School (MHB) Theodor Fontane Brandenburg an der Havel Germany; ^2^ Department of Anatomy and Cell Biology, Medical Faculty Martin Luther University Halle‐Wittenberg Halle (Saale) Germany

**Keywords:** alternative splicing, cancer, EBV, Herpes viruses, immune evasion, malignant transformation

## Abstract

The Epstein–Barr virus (EBV) belongs to the gamma subgroup of human herpesviruses. EBV is particularly lymphotropic regarding its latency, meaning that it preferentially targets lymphocytes as its immunological niche. During the establishment of the complex virus–host relationships, the virus influences the human host cell transcriptome inducing changes in RNA quantity and quality aided by a multitude of virally encoded molecules such as microRNAs, lncRNAs, peptides, and proteins. Quantitative changes in human gene expression of infected host cells are due to epigenetic mechanisms, such as altered promoter methylation patterns and altered microRNA‐ or lncRNA‐based post‐transcriptional gene regulation mechanisms. The quality of the human transcriptome is altered including changes in chemical RNA modifications and alterations in alternative splicing (AS) patterns. These molecular mechanisms contribute to the immune evasion of EBV, enhanced host cell proliferation and desensitization to apoptosis induction. These mechanisms are highly relevant to the process of malignant transformation of infected host cells, highlighting the tumorigenic potential of EBV. This review article addresses the role of AS in malignant transformation of EBV‐infected host cells. For this purpose, publicly available transcriptome datasets from EBV‐positive human Burkitt lymphomas (BL) were analyzed in an EBV‐copy number dependent manner. We identified 14 directly dysregulated splicing factors, predominantly components of the splicing machinery as well as trans‐acting splicing factors, with putative relevance for virus‐mediated alterations of host cell splicing patterns.

## Significance

This review focuses on the ability of the EBV to quantitatively and qualitatively alter the human transcriptome of the infected host cells. The effects on alternative splicing are highly relevant. A multitude of dysregulated human splicing factors that correlate with EBV copy numbers were identified. These splice factors are not only trans‐splicing factors but, in many cases also components of the splicing machinery itself. We identify new research approaches that may improve diagnosis and therapy of EBV‐induced tumor diseases.

**Abbreviations**
AS: alternative splicing
BL: Burkitt lymphoma
BLGSP: Burkitt Lymphoma Genome Sequencing project
CMV: cytomegalovirus
DESeq: differential gene expression analysis
DNA: deoxyribonucleic acid
EPV: Epstein–Barr Virus
FDR: false discovery rate
HHV: human herpesviruses
HSV: Herpes simplex virus
kbp: kilobase pairs
KSHV: Kaposi's sarcoma‐associated herpesvirus
LFC: Log2fold change
lncRNA: long non‐coding RNA
miR: microRNA
mRNA: messenger RNA
ncRNA: non‐coding RNA
NPC: nasopharyngeal carcinoma
RBP: RNA binding protein; RNA, ribonucleic acid
RNA‐Seq: RNA sequencing
snRNA: small nuclear RNA
VZV: varicella‐zoster virus

## 1 Overview of Human Herpesviruses and Their Key Characteristics

The eight different representatives of the human herpesviruses (HHV‐1 to 8) are enveloped viruses whose genome is composed of double‐stranded DNA. Their genome varies between different representatives, ranging from 125 kbp (kilobase pairs) in the varicella‐zoster virus (VZV, HHV‐3) to a 235 kbp large genome in the cytomegalovirus (CMV, HHV‐5) [1]. These large viral genomes encode a multitude of genes from which even more coding and non‐coding transcripts can be generated. The non‐coding transcripts, viral peptides and proteins serve to establish complex virus–host interactions. HHV infection rates can be very high within the global population (partially > 90%) associated with sometimes very long latency periods (with some HHVs, for the remaining host life) due to complex and highly specialized virus–host interactions.

After an initial infection, which frequently occurs in early childhood, the HHVs remain in a specific host cell population as an immunological niche for life. Among the HHVs the human Epstein–Barr virus (EBV, HHV‐4) shows the highest global infection rates of more than 90% causing mononucleosis, also known as kissing disease. Roseolaviruses (HHV‐6A/B), which are also acquired in early childhood and considered the cause of roseola are additionally characterized by a very high global seroprevalence rate of more than 90% [2]. VZV is another member of the HHV family with a very high seroprevalence. A closer examination of global VZV infection rates reveals strong regional differences. Very high VZV infection rates of more than 90% are observed only in a few regions of the developing countries, whereas significantly lower rates are detected in other regions, such as the European Union countries. This can be attributed to national vaccination policies or available vaccination options [3]. Another factor influencing global VZV seroprevalence is local climate. Evidence suggests that the local climate affects not only the seroprevalence but also the average infection age which may be linked to the course and severity of the VZV‐induced disease [4, 5]. The seroprevalence of cytomegalovirus (CMV, HHV‐5) varies considerably between industrialized (39%) and developing countries (> 90%). Herpes simplex virus 1 (HSV‐1, HHV‐1) shows infection rates differing between industrialized and developing countries: Europe (below 5%) and Africa (around 50%). In contrast, herpes simplex virus 2 (HSV‐2, HHV‐2) possesses only a low seroprevalence of approximately 13% [6, 7]. Kaposi's sarcoma‐associated herpesvirus (KSHV, HHV‐8) also exhibits extremely strong geographic variation in seroprevalence, ranging from highly endemic regions (50%–80% in sub‐Saharan African countries) to regions of low endemicity, such as industrialized countries (< 5%) [8].

Compared to other HHVs, EBV infection rates are very high, even in industrialized countries. A large amount of data on EBV life cycle‐dependent gene expression profiles and on the impact of viral molecules on infected host cells is available. Therefore, this review focusses on EBV as a representative example of other HHVs.

## 2 Establishment of an Effective Immunological Niche As the Result of a Complex Virus–Host Relationship

The high global HHV infection rates, across diverse climates and ethnic groups, and their ability to establish latencies lasting for life, presuppose intensive virus–host relationships that are highly adapted to the respective host cell types. In the past, research has focused on distinct virally encoded molecules such as viral proteins, peptides, long non‐coding transcripts, and virally encoded microRNAs [9, 10, 11]. However, the analysis of molecular virus–host interactions must also consider the transcriptome of the infected host cells. Virus‐induced alterations do not only cause simple quantitative up‐ and down‐regulations of host transcripts but also result in qualitative RNA changes, such as altered alternative splicing and putative RNA modifications.

These complex, multifactorial and multi‐level molecular virus–host interactions, e.g. in immune evasion, date back to an estimated 180‐200 million years of coevolution [12]. It is thought that this coevolution led to the splitting of herpesviruses into the three subgroups. The alpha‐subgroup includes HSV‐1/2 and VZV, which can establish latency in neurons of the host's sensory ganglia (HSV‐1 in the trigeminal ganglion; HSV‐2 in the sacral ganglia and VZV in the dorsal root ganglia, trigeminal ganglia, and cranial nerve ganglia [13, 14, 15]. Furthermore, a beta‐subgroup can be distinguished, whose representatives, CMV and the three roseoloviruses, establish a latency in monocytes/macrophages. CMV infects CD34‐positive hematopoietic precursor cells, as well as their CD34‐negative derivatives, monocytes and macrophages. Roseoloviruses establish latency in CD4‐positive T lymphocytes. All representatives of this beta subgroup are also known for establishing latency in the epithelial cells of the salivary glands [16, 17, 18].

EBV and KSHV are the two representatives of the gamma subgroup that are particularly lymphotropic for establishing latency, i.e. these viruses preferentially target lymphocytes. KSHV can infect B lymphocytes and endothelial cells of blood and lymphatic vessels for its latency [19]. EBV infects B, T, and NK lymphocytes as well as nasopharyngeal epithelial cells for its latency [20]. Furthermore, gamma herpesviruses possess an oncogenic potential [21].

From the perspective of the virus–host relationship, the coevolutionary division of herpesviruses into these three subgroups, with their own immunological niches, are highly important for their respective latency. In fact, herpesviruses do not pursue a “hit and run” strategy with rapid infection and spread but rather a “stay and low” strategy aimed at immune evasion and a reproductive phase when the host organism's immune system is weakened [22].

## 3 EBV Drives Complex Virus–Host Interactions Aiming at Reprogramming the Host Cell

Thanks to intensive research and advances in medicine and molecular biology, it has become possible to identify and characterize the complex interactions between viral molecules and host cells at a wide range of cell‐biological levels.

Molecular virus–host interactions serve different purposes depending on the respective life cycle phases (latent and lytic [23];) of the virus. These complex molecular virus–host interactions of EBV are best understood by examining the respective phases of the EBV life cycle separately, as strictly regulated gene expression occurs during each single phase. As an infectious particle, EBV consists of a linear double‐stranded genome enclosed within an icosahedral protein shell (capsid). This capsid is composed primarily of five viral proteins: the major component BCLF1 and the stabilizing factors BFRF3, BORF1, and BDLF1 as well as the portal protein for the viral capsid BBRF1 [24]. It follows an amorphous region called the tegument which also contains virally encoded proteins with a critical role for early‐stage infection as well as virus assembly and release from host cells [25, 26]. The outer lipid envelope of this virion is derived from the host cell from which the virion was released during the lytic phase. However, this lipid bilayer can contain up to 11 additional virus‐encoded glycoproteins. These glycoproteins can protrude from the membrane like spikes and might play highly specialized roles in the subsequent infection of cells [27]. EBV utilizes various glycoproteins to infect different target cells. To infect B cells, the virus utilizes its most abundant glycoprotein, gp350 (BLLF1) which interacts with CD21/CD35 on the B cells. Furthermore, the glycoproteins gH (BXLF2), gL (BKRF2), and gp42 (BZLF2) form a trimeric complex capable of binding to HLA‐II molecules on B cells [28]. Following the release from lysed B cells, the resulting EBV virions express strikingly small amounts or even no gp42. This contributes to high infection rates of oropharyngeal and nasopharyngeal epithelial cells. Epithelial cell infection utilizes the remaining gH/gL heterodimer, which can interact with EPHA2 on the epithelial cells [29].

The EBV life cycle can be divided into lytic and latent phases; the latent phase can be further subdivided into latency types 0, I, II, and III based on distinct gene expression profiles. Latency 0 represents the most restricted latency type. At this stage, the virus can persist silently, sometimes for the entire lifespan of the host. No protein‐coding viral genes are expressed, meaning that no viral peptides can be presented by the host cell's HLA molecules [30]. Among these viral non‐coding RNA (ncRNA) species expressed during latency 0 are the two long ncRNAs (lncRNAs) EBER1 and EBR2 and various EBV microRNAs (miRs). miRs (viral‐encoded or human ones) are small, 18–22 nt long, single‐stranded RNAs. They contain a binding sequence that is specific for their target (host) mRNAs. miR binding usually occurs within the 3′‐UTR leading to translational repression and mRNA decay after miR binding [31]. Within the EBV genome, the miR genes occur in clusters. Three clusters can be distinguished: the BHRF1 cluster (4 miRs), BART1 cluster (14 miRs), and BART2 cluster (26 miRs) [9]. During the latency 0 phase, the miRs in the two BART clusters 1 and 2 are expressed, while the BHRF1 cluster remains almost inactive.

During the latency I phase, only EBNA1 is expressed alongside the ncRNA species. In the latency II phase, LMP1, LMP2A, and LMP2B are expressed in addition to EBNA1 and the ncRNAs. The full spectrum of latent EBV genes is expressed only during latency III, comprising six EBV nuclear antigens (EBNA1/2/3A‐3C/LP), three latent membrane proteins (LMP1/2 A/2B), and the ncRNAs [23, 32].

The lytic phase of the EBV life cycle can also be subdivided. At the onset of the immediate‐early stage, the viral transcription factors BZLF1 (Zta) and BRLF1 (Rta) are expressed, which in turn drive the transcription of further target genes. During the subsequent early stage, certain enzymes involved in viral genome replication are primarily expressed. In the ensuing late stage, viral genes encoding for proteins involved in virion assembly are expressed, as well as lytic factors that ultimately trigger host cell lysis and the subsequent release of virions [33].

The virus achieves sufficient immune evasion in each of these life cycle phases through various parallel molecular strategies that induce additive effects. This illustrates virus host coevolution that results in highly specialized molecular virus–host interactions.

The sophisticated multifactorial strategy not only aims at immune evasion, like the downregulation of HLA‐Ia‐mediated antigen presentation upon the cell surface of infected host cells, e.g., by virus‐encoded microRNAs such as ebv‐miR‐BHRF1‐3, which downregulates TAP2 [34]. It further includes the downregulation of cellular stress markers, such as MICB as an important NKG2D ligand through ebv‐miR‐BART2‐5p [35] as well as MICA through ebv‐miR‐BART7 [36]. Furthermore, EBV miRs are involved in the downregulation of antiviral and pro‐inflammatory cytokines. For example, IFNL3 is downregulated by ebv‐miR‐BART7‐3p [10] and IFN type I signaling by ebv‐miR‐BART16 [37]. However, EBV‐encoded long non‐coding (lnc) transcripts also contribute to immune evasion. For example, it has been reported that the EBER1 and 2 transcripts stabilize the mRNAs of the anti‐inflammatory cytokine IL‐10 in EBV‐positive B cells [38]. These regulations via EBV‐encoded miRs and lncRNAs are important because these viral molecules even account for the limited number of molecules that are already expressed in the latent life cycle phases of the virus, as mentioned above [20].

During the lytic phases of the life cycle, most virally encoded peptides and proteins are produced. Many of these molecules exert immunomodulatory functions such as viral (v)IL‐10, which is encoded by the BCRF1 gene. It reduces the peptide presentation via HLA‐I and ‐II molecules from infected B lymphocytes and suppresses the secretion of several pro‐inflammatory and anti‐viral cytokines, such as IFN‐gamma, type I interferons, TNF‐alpha, IL‐2 and IL‐12. Furthermore, vIL‐10 effectively inhibits immune effector cells such as T and NK lymphocytes, macrophages and dendritic cells. Simultaneously, vIL‐10 stimulates infected B cells and promotes their proliferation [39].

By employing analogous molecular mechanisms, EBV can not only induce immune evasion of their infected host cells, e.g., using B cells as an immunological niche during their long latency period. In parallel, virus‐stimulated B cell proliferation and enhanced expression of anti‐apoptotic genes occur while pro‐apoptotic and anti‐proliferative cellular factors of the host cells are inhibited [40, 41]. These functional alterations of the infected host cells which are essential for the viral survival are also hallmarks of malignant transformation according to Douglas Hanahan and Robert A. Weinberg [42].

EBV can induce certain malignant tumor diseases in infected tissues. Nasopharyngeal carcinoma (NPC) and Burkitt lymphoma (BL) are EBV‐positive in more than 90% of cases. EBV is well known to contribute to their pathogenesis [20] by inducing genomic instability, accumulation of mutations, and epigenetic reprogramming [43, 44].

## 4 EBV‐Infection Alters Host Cell Transcriptome

The sheer number of regulatory molecules expressed by EBV inevitably results in a highly altered host cell transcriptome. These alterations cause not only quantitative changes in host cell mRNA abundance but also qualitative changes to the host cell transcriptome including altered splicing patterns of mRNAs [45].

The diverse causes of the altered host‐cell transcriptome induced by EBV begin already at the DNA level, for example, with the altered methylation or demethylation of promoter regions of coding genes. Current research focuses on characterizing the impact of EBV‐encoded molecules for altered DNA methylation patterns in the host cells. This includes methylation patterns of EBV DNA sequences, with relevance for life cycle‐dependent viral gene expression but also host cell DNA sequences [46].

These processes directly activate the JNK/AP‐1 signaling pathway, the NFκB signaling pathway, the MAPK pathway, and the Wnt/β‐catenin pathway, e.g., by silencing WNT inhibitors. This results in increased cell proliferation, cell migration, invasiveness, and reduced apoptosis inducibility in the EBV‐positive host cells – contributing to malignant transformation [47, 48, 49, 50, 51]. The alteration of these host cell pathways by EBV‐mediated DNA methylation is of great interest, since EBV simultaneously targets the same pathways with other EBV‐encoded molecules in parallel.

In particular, the non‐coding EBV transcripts expressed in all viral life phases, such as lncRNAs or miRs, are also relevant for the host cell transcriptome. It has been shown that several of the most abundant EBV miRs regulate the WNT pathway as well as the pathways of growth factors such as EGF and FGF, e.g., ebv‐miR‐BHRF1‐1, ebv‐miR‐BART1, ebv‐miR‐BART4, and ebv‐miR‐BART17 [9]. Other EBV‐miR interactions with relevance to the aforementioned signaling pathways include ebv‐miR‐BART1, which additionally activates the AKT and MAPK [52], and ebv‐miR‐BART13, which promotes NF‐κB signaling [53]. Furthermore, the lncRNAs EBER1 and EBER2 regulate the JNK and NF‐κB pathways [54]. In analogy to the multifactorial targeting of immunologically relevant host cell structures described above, these cell biological pathways are also targeted by multiple virally encoded molecules to induce host cell transformation required for optimal viral survival.

## 5 EBV‐Induced Effects Influence Not Only RNA Quantity but Also RNA Quality

Interestingly, these virally encoded molecules not only lead to a quantitative change in the host cell transcriptome, for example, by increasing or decreasing gene expression at the transcript level through promoter methylations or demethylations. Additionally, such mechanisms also act at the post‐transcriptional level by viral miRs, lnc‐RNAs, peptides, or proteins. But also, qualitative changes in the host cell transcriptome can be observed, including altered splicing patterns and chemical RNA modifications.

Among the most important chemical modifications of human transcripts are RNA methylation, acetylation, and pseudouridylation, which affect RNA properties such as transcript stability, splicing, and translation [55, 56]. Very few data on this topic are currently available in the literature. It has been shown that viral transcripts in EBV‐infected host cells are also modified by cellular enzymes of the host, e.g., RNA m6A methylation [57]. In fact, EBV even activates the cellular m6A modification to promote cell survival and virus production during the lytic cycle [58]. No data are yet available in the literature on other RNA modifications in the context of EBV infection.

A very exciting research area, which will be addressed as a thematic focus in the following, is the impact of virus‐modified alternative splicing patterns within the EBV‐infected host cells. In addition to constitutive splicing, which is the simple splicing of introns followed by exon ligation during mRNA maturation, alternative splicing (AS) is further distinguished. Besides simple intron retention, four other mechanisms of AS, namely exon skipping, alternative 5′ splice site, alternative 3′ splice site, and coordinated exon splicing, have been identified. These mechanisms result in a high number of diverse splice variants [45]. For AS, in addition to the cis ‐acting elements on the mRNA sequence itself, trans‐regulatory factors play a particularly important role. These trans‐regulatory factors include certain RNA‐binding proteins (RBPs) that either promote or suppress the AS of an exon, acting as enhancers or silencers [45]. Several families of RBPs are known, some of which even control AS in a tissue‐specific manner. For example, the SR protein family, which includes among others SRSF2, tends to act as enhancers. On the other hand, the hnRNP family members are more commonly known to be silencers. Representatives of the CELF, RBOX, NOVA, MBNL, and PTBP families influence AS in a tissue‐specific and in some cases in an age‐dependent way, e.g. in fetal tissues [59, 60, 61, 62, 63].

Given these numerous trans‐regulatory splice factors, it is easy to guess that the previously mentioned EBV‐induced molecular alterations in the host cell transcriptome also dysregulate some of these factors.

This study aims to examine in detail the impact of EBV on the alternative splicing (AS) of the host cell. Indeed, this influence of EBV on the host cell begins with the expression of the lncRNAs EBER1 and EBER2, which are themselves capable of altering the alternative splicing of multiple host cell mRNAs. Using RNA sequencing analysis combined with EBER1/2 overexpression, Pimienta et al. (2015 [64];) identified 379 mRNAs that underwent statistically significant changes in alternative splicing. This result could be explained by other observations. For instance, Lee et al. (2012) demonstrated that the EBV lncRNA EBER1 is a direct binding partner of the trans‐splicing factor hnRNPD (AUF1) [65] which can also influence the RNA stability of other host cell transcripts. From this regulatory axis, further additional indirect effects on the host cell transcriptome might arise. In the same study, two additional hnRNPs, namely hnRNP A1 and hnRNP A2/B1, were also identified as EBER1 interaction partners. For EBER2, it was at least demonstrated that it can bind certain RBPs (NONO, SFPQ, RBM14) known to be involved in the regulation of alternative splicing as trans‐acting factors [66]. Whether these interactions also result in an altered AS remains to be investigated.

Interestingly, the EBV protein SM, which is involved in host cell lysis, is also thought to be capable of influencing the AS of certain host cell mRNAs, e.g., STAT1 mRNA [67]. Furthermore, Chua et al. (2022) demonstrated that the EBV proteins EBNA1, Zta, and Rta influence the AS of TSG101 pre‐mRNA, thereby contributing even to malignant transformation [68]. These specific interactions between SM and EBER1 with the host cell AS were previously summarized in an excellent review article by Boudreault et al. (2019 [69];). The authors also included additional molecular interactions that lead to alterations in the host cell's alternative splicing (AS), derived from many other viruses. Their conclusion aligns with our own hypothesis: the changes in host cell AS reflect intense virus–host interactions.

Another important observation was reported by Park et al. (2018 [70];). Indeed, certain cellular trans‐splicing factors (SRSF2, SRSF3, and Son) and RBPs (ALY, NXF1, and Y14) can be recruited to so‐called virus‐induced nodular structures (VINORCs) during EBV replication. Consequently, competitive effects on host cell transcripts and their AS are conceivable. These VINORCs form during the late lytic phase and are located at the periphery of viral replication compartments [70].

A comprehensive study, which addresses the central theme of this present analysis, was conducted by Armero et al. (2017 [71];). In this study, RNA‐Seq datasets from 26 EBV‐positive gastric adenocarcinoma tissues from The Cancer Genome Atlas (TCGA) were compared with corresponding EBV‐negative samples and transcripts exhibiting altered alternative splicing (AS) were identified. However, this study has a limitation that we aim to avoid in our own analysis. Specifically, our study takes EBV copy number into account during the database analysis. Armero et al. (2017 [71];) identified 33 AS‐related genes as differentially expressed when comparing EBV‐positive and EBV‐negative gastric tumors, a finding that is highly relevant to our own results.

Furthermore, Homa et al. (2013 [72];) report a significant shortening of 3’ UTRs and the exclusion of cassette exons in EBV‐transformed cells compared to uninfected B cells. However, these measurements were not performed using RNA sequencing analysis but by cDNA microarray analyzes (in line with the possibilities of the time), which detect sequence alterations only to a very limited extent depending on the probes present on the chip surface.

## 6 Analysis of a TCGA Dataset From Burkitt Lymphoma Patients Regarding a Putative Influence of EBV on Human Splicing Factors

In summary, findings from recent studies demonstrate that EBV influences AS of infected host cells. Preliminary insights into the underlying molecular regulatory mechanisms are already available, which include the dysregulation of human trans‐splicing factors. However, none of the existing studies specifically addressed the question of how EBV copy numbers impact this altered AS. We propose that the higher EBV copy number in an infected host cell the more dysregulated is the host cell transcriptome. Naturally, EBV copy number correlates with the virus's life cycle stages, with copy numbers rising drastically from the latent to the lytic phase [73, 74]. Yet, even during the latent phase, EBV copy numbers in infected host cells can vary by a factor of 100. To explore this in greater depth, EBV‐positive Burkitt lymphoma samples were analyzed. The data evaluation specifically examined host cell gene dysregulation in relation to EBV copy numbers. Furthermore, following these initial data analyzes, particular attention was paid to dysregulated host cell genes with putative functional relevance for alternative splicing.

Thus, also competitive consequences for human splicing events could be possible. In a study by Evelyne Manet et al., 2021 [75] it was shown that the AS patterns in B cells change as early as day 1 after EBV infection.

Therefore, we aimed to investigate the impact of EBV on human splicing factors in an in‐silico TCGA data analysis in a data set derived from BL (Burkitt Lymphoma Genome Sequencing project (BLGSP), a project of the Cancer Genome Characterization Initiative (Project ID: CGCI‐BLGSP; Dataset Accession: pht001132.v19.p6).

In fact, a nasopharyngeal carcinoma (NPC) transcriptome dataset also exists. However, this dataset contains significantly fewer specimens as the BL data set. NPC itself is inherently much more heterogeneous. Its RNA sequencing data could represent a diverse mixture of malignant nasopharyngeal epithelial cells from various locations along with infiltrating cells (e.g. immune effector cells or connective tissue cells like fibroblasts). Whereas BL originates from distinct B cells, which ensures better comparability between different patient specimens.

Also of relevance is the fact that BL cells are primarily in the EBV latency I phase [76], meaning only a limited number of EBV‐encoded molecules are expressed. Nevertheless, high differences in EBV copy numbers may exist among infected cells, which very likely leads to corresponding gene dosage effects, for instance, regarding the downregulation of immunologically relevant host cell molecules or effects on the alternative splicing (AS) machinery.

Following malignant transformation, BL cells can harbor higher EBV copy numbers than EBV‐positive B cells [77]. This fact has not yet been effectively considered in the TCGA data analyzes of EBV‐positive tumors of the summarized studies.

For an analysis, the following approximations had to be made: BLs are more than 90% EBV positive [75]. Furthermore, these datasets are only mapped against the human transcriptome and do not contain information on viral transcripts. Therefore, four human genes were selected whose transcript copy number is known. These were correlated with the respective EBV copy number within the infected host cells. The following four human genes were selected for this purpose: NF‐κB, HIF1α, MYC, AICDA [78, 79, 80, 81]. Separate analyzes were performed for each of these four genes, using the 10% highest and 10% lowest expressors for each of these genes from the BL specimen. Dysregulated genes (up‐ and downregulation) were identified by calculation of statistical significance (false discovery rate (FDR) < 0.05) (Figure 1A–D) followed by Log2fold change (LFC) calculation (cutoffs LFC < 0.5 and LFC > 2.0) of the previously statistically significant dysregulated genes visualized as heat maps (Figure 2A–D).

**Figure 1:**
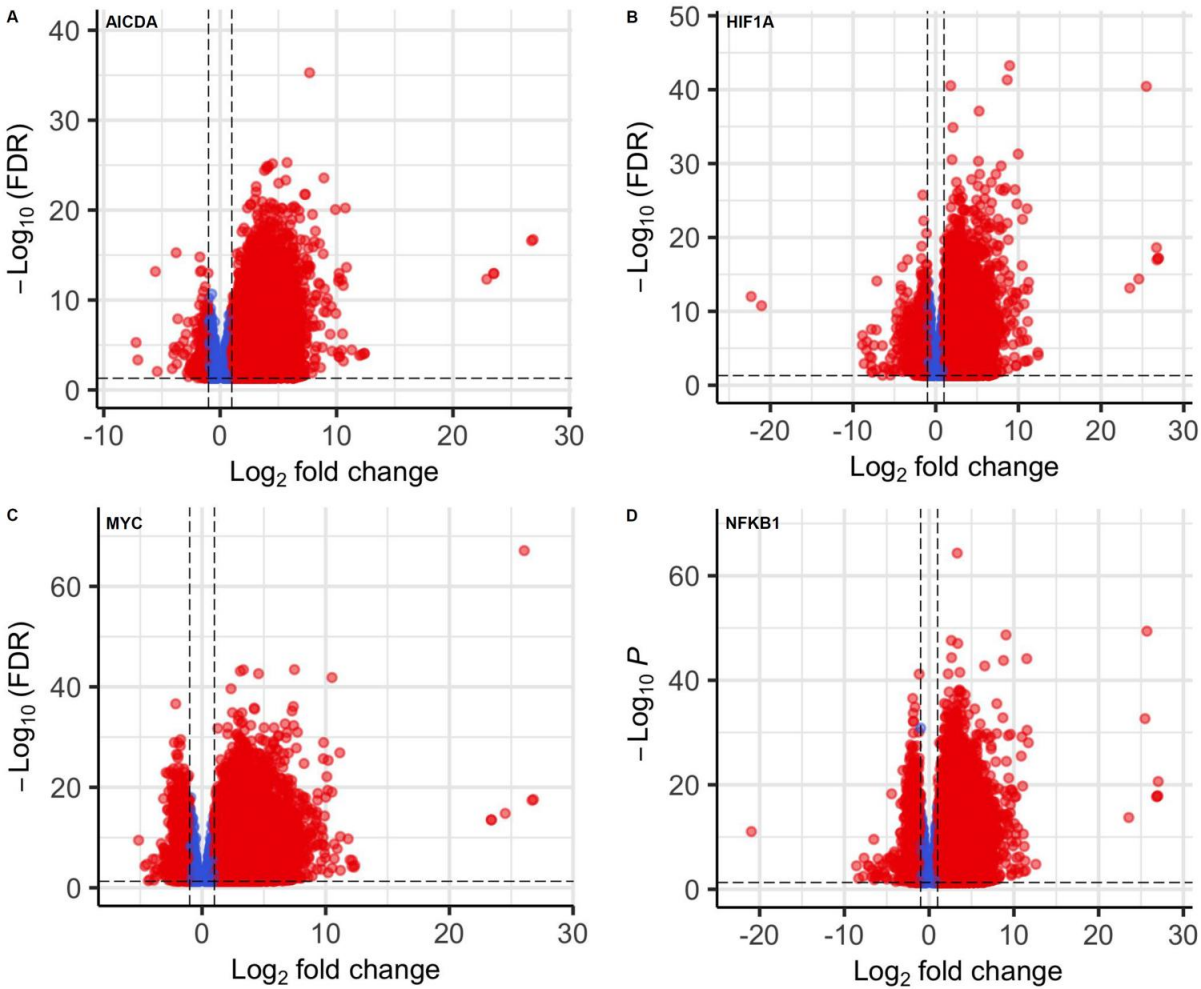
Volcano plots (A–D) show the dysregulated genes after RNA sequencing in the context of the EBV copy number‐correlated genes AICDA (A), HIF1α (B), MYC (C), and NFKB1 (D) filtered for highest expression (0.9 quantile) and lowest expression (0.1 quantile) within each of the four genes, with an FDR ≤ 0.05 set in DESeq. 2 analysis. Each dot indicates a gene, whereas red dots represent genes within the set LFC‐thresholds, bule dots only meet an FDR ≤ 0.05 but not the set LFC‐thresholds (high: LFC ≥ 2; low: LFC ≤ 0.5). (low expresser group *n* = 20, high expresser group *n* = 20) visualized in R (Version R. Version 4.5.3).

**Figure 2:**
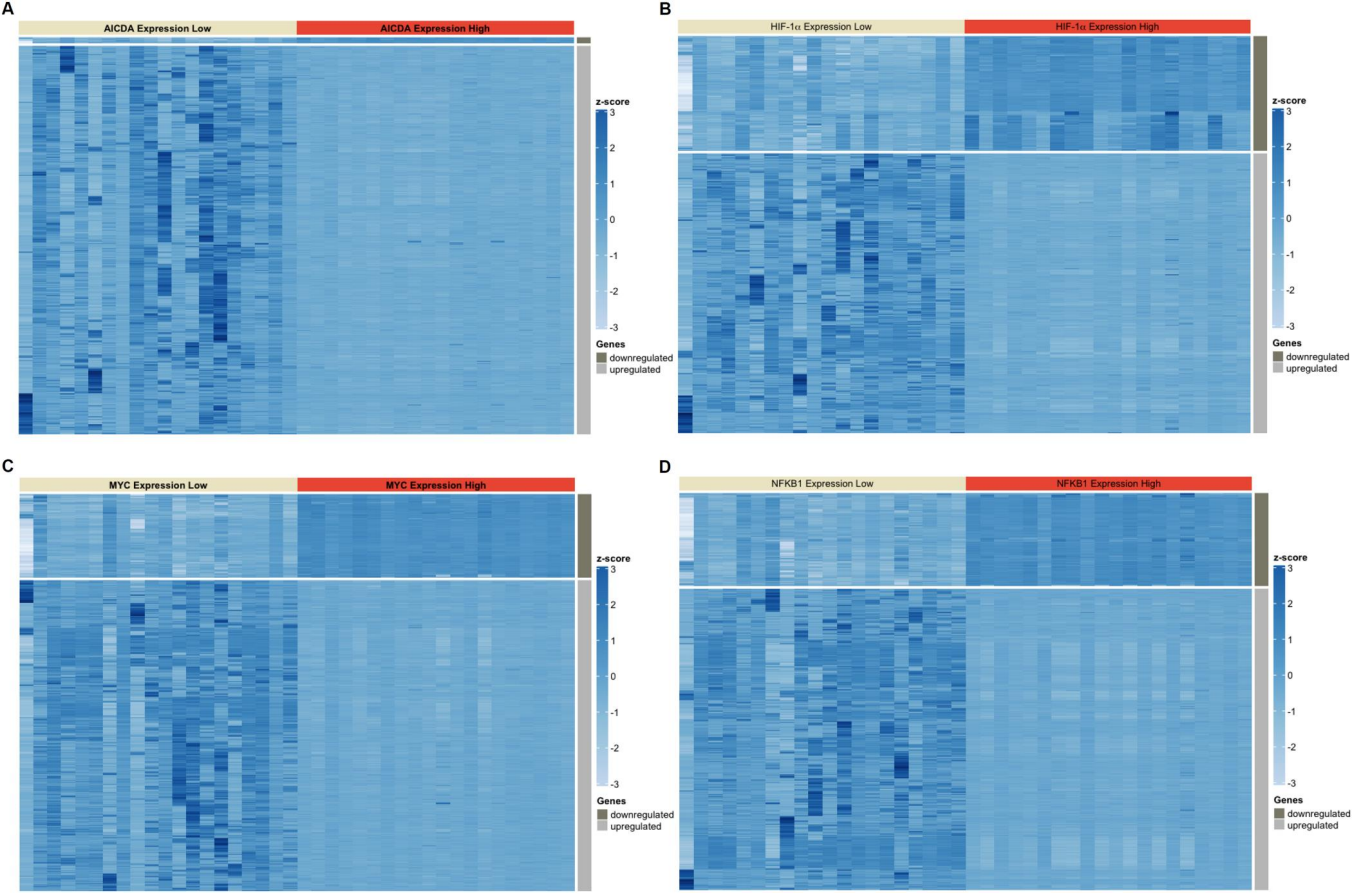
The differential gene expression pattern of statistically significantly dysregulated genes of the AICDA (A), HIF1α (B), MYC (C), and NFKB1 (D) analyzes visualized as heatmaps show the expression pattern of the two quantile‐defined groups, low (LFC ≤ 0.5) versus high (LFC ≥ 2) within each gene of interest (FDR ≤ 0.05); expression patterns were variance‐stabilization transformed (VST) and *z*‐score normalized (low‐*n* = 20, high‐*n* = 20) (Version R. Version 4.5.3).

Afterwards, Venn diagrams were used to identify those genes that were either statistically significantly upregulated (*n* = 633) or downregulated (*n* = 27) in all four analysis groups – taking into account the cut‐off values used for the respective LFCs. (Figure 3A,B).

**Figure 3:**
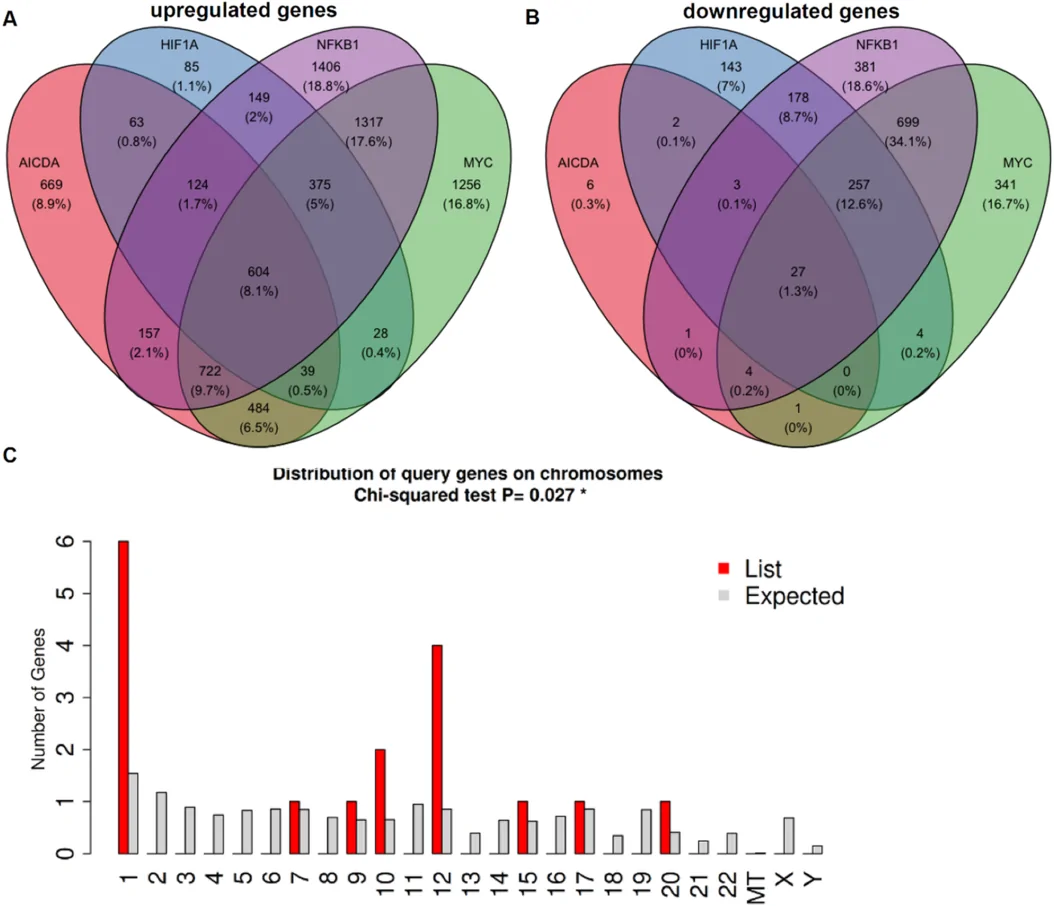
Identification of mutually shared dysregulated genes within all four genes of interest (A‐B) as Venn diagrams. Venn diagrams display common shared genes within two defined groups high (A) versus low (B), comparing all four genes (AICDA, HIF1α, MYC and NFKB1) within these groups. Identification of 633 common shared statistically significantly upregulated genes (LFC ≥ 2; FDR ≤ 0.05) and 27 statistically significantly downregulated genes (LFC ≤ 0.5; FDR≤ 0.05). All dysregulated splice factors and RBPs (Table 1) were submitted to ShinyGO 0.85.1; and an annotated localization list was calculated against the expected statistical chromosomal distribution of the genes in the human genome (gray = expected bars) and background list (self‐submitted). Difference in distribution was chi‐squared tested with *p* = 0.027; figures A and B created in R (Version R. Version 4.5.3), C created using ShinyGO 0.85.1.

For the statistically significantly dysregulated genes, the known functions were manually assigned using the website https://www.ncbi.nlm.nih.gov/gene. This analysis revealed that 14 of these genes are directly involved in RNA splicing, while four additional genes encode for RBPs ‐ with PRMT8 in particular, being also directly involved in the RNA methylations (Table 1; the full list is available as Supporting Information: Table S1).

**Table 1:** Results from the functional annotation of differentially expressed genes related to transcriptional processes.

Gene symbol	Function	Upregulated/downregulated in BL data set	Gene locus	Expression in tumor diseases
A1CF	The gene product has three non‐identical RNA recognition motifs and belongs to the hnRNP R family of RNA‐binding proteins. It has been proposed that this complementation factor functions as an RNA‐binding subunit and docks APOBEC‐1 to deaminate the upstream cytidine. Studies suggest that the protein may also be involved in other RNA editing or RNA processing events.	upregulated	10 (10q11.23)	upregulated in breast cancer, lung cancer, renal cell carcinoma, hepatocellular carcinoma, endometrial cancer, and glioma [82, 83, 84, 85, 86, 87, 88, 89, 90]
HNRNPF	This gene belongs to the subfamily of ubiquitously expressed heterogeneous nuclear ribonucleoproteins (hnRNPs). The hnRNPs are RNA binding proteins that complex with heterogeneous nuclear RNA (hnRNA). These proteins are associated with pre‐mRNAs in the nucleus and regulate alternative splicing, polyadenylation, and other aspects of mRNA metabolism and transport.	downregulated	10 (10q11.21)	upregulated in Gastric cancer, pancreatic cancer; breast cancer [91, 92, 93]
RNU4‐1	Predicted to enable U6 snRNA binding activity. Predicted to be involved in formation of quadruple SL/U4/U5/U6 snRNP and spliceosomal tri‐snRNP complex assembly. Predicted to be part of U4 snRNP and U4/U6 x U5 tri‐snRNP complex.	upregulated	12 (12q24.23)	upregulated in prostate cancer [94]
RNU4‐2	Predicted to enable U6 snRNA binding activity. Predicted to be involved in formation of quadruple SL/U4/U5/U6 snRNP and spliceosomal tri‐snRNP complex assembly. Predicted to be part of U4 snRNP and U4/U6 x U5 tri‐snRNP complex.	upregulated	12 (12q24.23)	upregulated in colon cancer and in prostate cancer [94, 95, 96]
RNU5B‐1	Predicted to be involved in formation of quadruple SL/U4/U5/U6 snRNP and spliceosomal tri‐snRNP complex assembly. Predicted to be part of U4/U6 x U5 tri‐snRNP complex and U5 snRNP.	upregulated	15 (15q22.31)	n.d.
RNU5D‐1	Predicted to be involved in formation of quadruple SL/U4/U5/U6 snRNP and spliceosomal tri‐snRNP complex assembly. Predicted to be part of U4/U6 x U5 tri‐snRNP complex and U5 snRNP.	upregulated	1 (1p34.1)	n.d.
RNU6ATAC/U6atac snRNA	Predicted to enable U4atac snRNA binding activity and pre‐mRNA 5′‐splice site binding activity. Predicted to be involved in mRNA 5′‐splice site recognition and spliceosomal tri‐snRNP complex assembly. Predicted to be part of U6atac snRNP.	upregulated	9 (9q34.2)	upregulated in Prostate Cancer, Pituitary neuroendocrine tumors (PitNETs) [97, 98, 99]
RNVU1‐18	Predicted to enable pre‐mRNA 5′‐splice site binding activity. Predicted to be involved in mRNA 5′‐splice site recognition. Predicted to be part of U1 snRNP.	upregulated	1 (1q21.1)	n.d.
RNVU1‐2	Predicted to enable pre‐mRNA 5′‐splice site binding activity. Predicted to be involved in mRNA 5′‐splice site recognition. Predicted to be part of U1 snRNP. [	upregulated	1 (1q21.2)	differential dispersion in lung adenocarcinoma [100]
RNVU1‐28	Predicted to enable pre‐mRNA 5′‐splice site binding activity. Predicted to be involved in mRNA 5′‐splice site recognition. Predicted to be part of U1 snRNP.	upregulated	1 (1q21.1)	Upregulated in high‐risk‐prostatecancer [94, 101]
RNVU1‐31	Predicted to enable pre‐mRNA 5′‐splice site binding activity. Predicted to be involved in mRNA 5′‐splice site recognition. Predicted to be part of U1 snRNP.	upregulated	1 (1q21.1)	n.d.
RNVU1‐7	Predicted to enable pre‐mRNA 5′‐splice site binding activity. Predicted to be involved in mRNA 5′‐splice site recognition. Predicted to be part of U1 snRNP.	upregulated	1 (1q21.2)	n.d.
SNRPB	The protein encoded by this gene is one of several nuclear proteins that are found in common among U1, U2, U4/U6, and U5 small ribonucleoprotein particles (snRNPs). These snRNPs are involved in pre‐mRNA splicing, and the encoded protein may also play a role in pre‐mRNA splicing or snRNP structure.	downregulated	20 (20p13)	upregulated in ESCC [102], upregulated in HCC [103]
SRRM4	SRRM4 promotes alternative splicing and inclusion of neural‐specific exons in target mRNAs.	upregulated	12 (12q24.23)	upregulated in breast cancer and Small cell lung cancer [104, 105]
MYH4	Enables double‐stranded RNA binding activity. Involved in muscle contraction. Located in myofibril.	upregulated	17 (17p13.1)	upregulated in colon adenocarcinoma [106]
PRMT8	Arginine methylation is a widespread posttranslational modification mediated by arginine methyltransferases, such as PRMT8. Arginine methylation is involved in a number of cellular processes, including DNA repair, RNA transcription, signal transduction, protein compartmentalization, and possibly protein translation	upregulated	12 (12p13.32)	upregulated in breast, ovarian and cervical cancer; upregulated in Lung cancer [107, 108]

*Note:* Upregulation and down regulation defined with DESeq. 2 analyzed RNA sequencing data; function and gene locus was identified via NCBI – Genes.

The results regarding RNA‐splicing factors are of special importance for the initially proposed influence of EBV on AS within the host cell. Among them are not only isolated trans‐regulatory splicing factors ‐ such as A1CF, HNRNPF, and SRRM4, but also numerous core elements with direct relevance to the splicing machinery itself: RNU4‐1, RNU4‐2, RNU5B‐1, RNU5D‐1, RNU6ATAC, RNVU1‐18, RNVU1‐2, RNVU1‐28, RNVU1‐31, RNVU1‐7, and SNRPB (Table 1).

We identified certain dysregulated snRNAs with relevance to the major spliceosome (U2‐dependent spliceosome; specifically RNU4‐1, RNU4‐2, RNU5B1, and RNU5D‐1) and of snRNAs relevant to the minor spliceosome (U12‐dependent spliceosome; specifically RNU6AT). These findings suggest that, in addition to alternative splicing, constitutive splicing could also be dysregulated by EBV infection, even at defined and special sites. However, these observations require further in‐depth analyzes and validations.

The majority of these 14 dysregulated splice factors and 2 additional dysregulated general RBPs of Table 1 are encoded on chromosome 1, which statistically significantly exceeded the statistical expectations for gene encoding on this chromosome (*p* = 0.027; Figure 3C).

Interestingly, independent of EBV infection, many of these RBPs and splicing factors have already been identified as dysregulated in various tumor diseases (Table 1). These data support the hypothesis that EBV infections indeed have a high relevance for AS. The viral copy number alone within infected host cells (even in different tissues) leads to altered gene expression of numerous RBPs, with functional relevance for AS. This could be important for the molecular processes of malignant transformation. More precise insights into EBV‐induced alterations of the host cell transcriptome could not only yield mechanistic insights into virus–host interactions but also improve diagnosis or even treatment and prognosis of EBV‐induced tumor diseases.

However, this indirect approach has certain limitations and relies on simplifications that should be mentioned.

The optimal procedure would be to utilize specific EBV transcripts for sample‐specific normalization factors (also to minimize sample differences in quality and individual gene expression) to estimate EBV copy numbers in EBV‐positive tumors. Notably, the lncRNAs EBER1 and EBER2, as well as certain EBV miRs, are expressed throughout all phases of the EBV life cycle; while they could theoretically be identified from the original RNA sequencing data, this information is no longer retrievable from the TCGA files, which have already been mapped to the human transcriptome. The applied indirect approach using other human marker genes has serious limitations. Genes such as NF‐κB, HIF1α, MYC, and AICDA are frequently dysregulated and mutated even in EBV‐negative tumor diseases. This can also occur in EBV‐positive tumors independent of the virus itself.

Another aspect is that these factors can influence one another; for instance, NF‐κB can induce the expression of HIF1α [109]. Since both factors are hypothesized to correlate with EBV copy numbers, these mutual dependencies should have only a minimal confounding effects.

Further in silico data analyzes based on the identified dysregulated factors could lead to the identification of differentially spliced host cell mRNAs. However, considering the many simplifications in our analysis, further in silico analyzes should be based upon dysregulated targets that have been validated using molecular biological methods (e.g., qPCR and Western blot).

## 7 Material and Methods

### 7.1 Data Set

For the in silico analysis RNA sequencing (RNA‐Seq) data of BL primary tumors were obtained from the Burkitt Lymphoma Genome Sequencing project (BLGSP), a project of Cancer Genome Characterization Initiative (Project ID: CGCI‐BLGSP; Dataset Accession: pht001132.v19.p6). The STAR‐aligned gene level‐counts were drawn from open accessible Genomic Data Commons (GDC) Data Portal in April of 2026 (https://portal.gdc.cancer.gov). The initial query contained 283 RNA‐sequencing files. After filtering for primary tumor sets only, a total of 257 cases was extracted. Removing duplicates in patients left 196 unequipped cases for differential gene expression analysis (DESeq).

## 8 Data Analysis

Differential expression analysis (DE) and visualization of further downstream analysis were conducted in R (R. Version 4.5.3) using Biconductor implementations (release Version: 3.22; BiocManager 1.30.27) as well as ggplot2 (V 4.0.3). Data sets for DE were downloaded using TCGAbiolinks (V 2.38.0) [110]. To identify transcriptional changes within the BLGSP‐cases, four DE analysis were performed each sampling one of the four virus‐correlating genes: AICDA, MYC, HIF1α, and NF_K_B1. Within each group the 10% highest raw expression were compared with the 10% lowest raw expression of the 196 samples. Whereas the 0.1 quantile defining the lowest 10% (*n* = 20) and the 0.9 quintile defining the highest 10% (*n* = 20) of raw expression counts of each gene, yielding 40 samples per analysis. The genes were identified by their gene symbols.

### 8.1 Differential Expression Analysis

The raw filtered counts were handed to DE analysis using DESeq. 2 (V 1.50.2) [111]. Genes were defined as differently expressed if they had an FDR of 0.05, for upregulation an LFC greater than 2 and for downregulated genes an LFC lower than 0.5. Expression patterns were visualized applying the same cut‐offs as in the underlying DE.

### 8.2 Heatmaps

Variance‐stabilization transformation (VST) was applied and z‐score normalization for visualization of expression pattern between samples (ComplexHeatmap; V 2.26.1) [112].

### 8.3 Intersection Analysis

The result of DESeq. 2 was displayed in Venn diagram, sorting for commend shared differential expressed genes (VennDetail; V 1.26.1) within the group defining genes.

Functional Enrichment Analysis

Functional enrichment analysis was based on the intersection analysis. Here functions were annotated manually using NCBI (https://www.ncbi.nlm.nih.gov/gene), using the gene filter. As an additional filter, transcriptome related genes were selected and submitted to ShinyGO 0.85.1 (https://bioinformatics.sdstate.edu/go/) [113] for chromosomal location analysis. The gene list was submitted with a corresponding background list, containing all genes in the initial data set obtained from GDC.

## 9 Conclusions

Immediately after infection, the EBV virus begins not merely to manipulate individual target genes or even specific cellular pathways within the infected human host cells. For this, it uses its multitude of virus‐encoded molecules to induce an extensive reprogramming of the host cell across multiple regulatory levels, including the genome, transcriptome, and proteome. This reprogramming results in, among other things, increased immune evasion, cell proliferation, and resistance to apoptosis. Yet due to its molecular interaction possibilities it can additionally and inevitably pave the way for the complete malignant transformation of the host cell (summarized in Figure 4). These results are intended not only to support the hypothesis but also to encourage further scientific research to dedicate itself to this still insufficiently investigated molecular biological topic.

**Figure 4:**
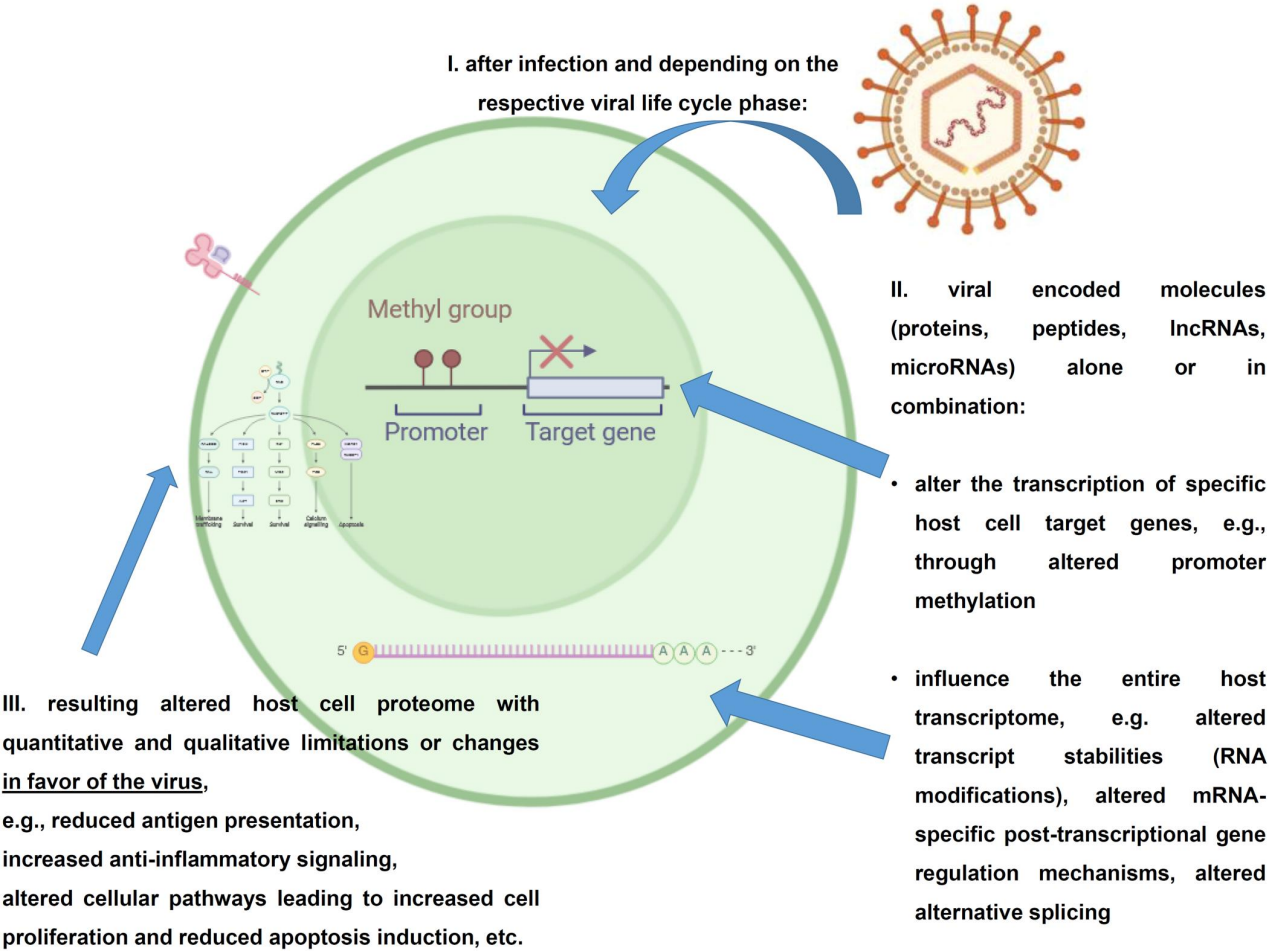
Schematic overview illustrating the key effects of EBV on the host cell transcriptome (created with BioRender.com).

## Author Contributions

Simon Jasinski‐Bergner designed the project, and the first draft of the manuscript was written by Simon Jasinski‐Bergner and Louise Wallot Experimental work, data collection, analyzes, interpretations, and visualizations were performed by Simon Jasinski‐Bergner, Louise Wallot, and Tim Hohmann The figures and tables were created by Louise Wallot as well as Simon Jasinski‐Bergner The revised final version of the manuscript was created by Louise Wallot, Tim Hohmann, Urszula Hohmann, Faramarz Dehghani, Olaf Grisk, and Simon Jasinski‐Bergner, which was approved by all authors. All authors have read and agreed to the published version of the manuscript.

## Supporting information


Supporting File

## Data Availability

The data that support the findings of this study are openly available in CGCI‐BLGSP at https://portal.gdc.cancer.gov, reference number pht001132.v19.p6.

## References

[1] C. J. Houldcroft , “Human Herpesvirus Sequencing in the Genomic Era: The Growing Ranks of the Herpetic Legion,” Pathogens 8, no. 4 (2019): 186, 10.3390/PATHOGENS8040186. PMC6963362 31614759

[2] M. Pippin and G. Laws , “A Classic Presentation of Roseola Infantum,” Cureus 16, no. 1 (2024): 52504, 10.7759/CUREUS.52504. PMC10874238 38371053

[3] A. Nardone , F. de Ory , M. Carton , et al., “The Comparative Sero‐Epidemiology of Varicella Zoster Virus in 11 Countries in the European Region,” Vaccine 25, no. 45 (2007): 7866–7872, 10.1016/j.vaccine.2007.07.036. 17919788

[4] S. W. P. L. Daulagala and F. Noordeen , “Epidemiology and Factors Influencing Varicella Infections in Tropical Countries Including Sri Lanka,” VirusDisease 29, no. 3 (2018): 277–284, 10.1007/s13337-018-0459-z. PMC6111959 30159361

[5] N. Farhat , D. El Safadi , J. Massoud , and S. Khalife , “Seroprevalence and Vaccination Determinants of Varicella Zoster Virus Among Pediatric and Adolescent Populations in Northern Lebanon,” Vaccines 13, no. 11 (2025): 1166, 10.3390/VACCINES13111166. PMC12656992 41295539

[6] S. de Sanjose , G. Mbisa , S. Perez‐Alvarez , et al., “Geographic Variation in the Prevalence of Kaposi Sarcoma‐Associated Herpesvirus and Risk Factors for Transmission,” Journal of Infectious Diseases 199, no. 10 (2009): 1449–1456, 10.1086/598523. 19351262

[7] C. James , M. Harfouche , N. J. Welton , et al., “Herpes Simplex Virus: Global Infection Prevalence and Incidence Estimates, 2016,” Bulletin of the World Health Organization 98, no. 5 (2020): 315–329, 10.2471/BLT.19.237149. PMC7265941 32514197

[8] L. M. Butler , G. Dorsey , W. Hladik , et al., “Kaposi Sarcoma‐Associated Herpesvirus (KSHV) Seroprevalence in Population‐Based Samples of African Children: Evidence for at Least 2 Patterns of Kshv Transmission,” Journal of Infectious Diseases 200, no. 3 (2009): 430–438, 10.1086/600103. PMC3975590 19534596

[9] T. Hohmann , U. Hohmann , F. Dehghani , O. Grisk , and S. Jasinski‐Bergner , “Analyzing the Impact of the Highest Expressed Epstein‐Barr Virus‐Encoded microRNAs on the Host Cell Transcriptome,” International Journal of Molecular Sciences 25, no. 14 (2024): 7838, 10.3390/ijms25147838. PMC11276978 39063079

[10] J. Blümke , M. Bauer , C. Vaxevanis , et al., “Identification and Characterization of the Anti‐Viral Interferon Lambda 3 as Direct Target of the Epstein‐Barr Virus Microrna‐Bart7‐3P,” Oncoimmunology 12, no. 1 (2023): 2284483, 10.1080/2162402X.2023.2284483. PMC10732682 38126030

[11] S. Jasinski‐Bergner , O. Mandelboim , and B. Seliger , “Molecular Mechanisms of Human Herpes Viruses Inferring With Host Immune Surveillance,” Journal for Immunotherapy of Cancer 8, no. 2 (2020): e000841, 10.1136/JITC-2020-000841. PMC7333871 32616556

[12] D. J. McGeoch , S. Cook , A. Dolan , F. E. Jamieson , and E. A. R. Telford , “Molecular Phylogeny and Evolutionary Timescale for the Family of Mammalian Herpesviruses,” Journal of Molecular Biology 247, no. 3 (1995): 443–458, 10.1006/JMBI.1995.0152. 7714900

[13] N. M. Thellman and S. J. Triezenberg , “Herpes Simplex Virus Establishment, Maintenance, and Reactivation: In Vitro Modeling of Latency,” Pathogens 6, no. 3 (2017): 28, 10.3390/PATHOGENS6030028. PMC5617985 28644417

[14] A. A. Gershon , J. Breuer , J. I. Cohen , et al., “Varicella Zoster Virus Infection,” Nature Reviews Disease Primers 1 (2015): 15016, 10.1038/NRDP.2015.16. PMC5381807 27188665

[15] L. Zerboni , C. C. Ku , C. D. Jones , J. L. Zehnder , and A. M. Arvin , “Varicella‐Zoster Virus Infection of Human Dorsal Root Ganglia In Vivo,” Proceedings of the National Academy of Sciences 102, no. 18 (2005): 6490–6495, 10.1073/PNAS.0501045102. PMC1088374 15851670

[16] A. Jarvis , “Mechanisms of Human Cytomegalovirus Persistence and Latency,” Frontiers in Bioscience 7 (2002): d1575, 10.2741/JARVIS. 12045013

[17] S. Pantry and P. Medveczky , “Latency, Integration, and Reactivation of Human Herpesvirus‐6,” Viruses 9, no. 7 (2017): 194, 10.3390/V9070194. PMC5537686 28737715

[18] F. Miyake , T. Yoshikawa , H. Sun , et al., “Latent Infection of Human Herpesvirus 7 in CD4(+ ) T Lymphocytes,” Journal of Medical Virology 78, no. 1 (2006): 112–116, 10.1002/JMV.20511. 16299718

[19] S. M. Akula , P. P. Naranatt , N. S. Walia , F. Z. Wang , B. Fegley , and B. Chandran , “Kaposi's Sarcoma‐Associated Herpesvirus (Human Herpesvirus 8) Infection of Human Fibroblast Cells Occurs Through Endocytosis,” Journal of Virology 77, no. 14 (2003): 7978–7990, 10.1128/JVI.77.14.7978-7990.2003. PMC161913 12829837

[20] M. Bauer , S. Jasinski‐Bergner , O. Mandelboim , C. Wickenhauser , and B. Seliger , “Epstein‐Barr Virus‐Associated Malignancies and Immune Escape: The Role of the Tumor Microenvironment and Tumor Cell Evasion Strategies,” Cancers 13, no. 20 (2021): 5189, 10.3390/cancers13205189. PMC8533749 34680337

[21] R. F. Ambinder , “Gammaherpesviruses and “Hit‐And‐Run” Oncogenesis,” American Journal of Pathology 156, no. 1 (2000): 1–3, 10.1016/S0002-9440(10)64697-4. PMC1868625 10623645

[22] B. Grinde , “Herpesviruses: Latency and Reactivation ‐ Viral Strategies and Host Response,” Journal of Oral Microbiology 5, no. 1 (2013): 22766, 10.3402/JOM.V5I0.22766. PMC3809354 24167660

[23] R. J. Tierney , C. D. Shannon‐Lowe , L. Fitzsimmons , A. I. Bell , and M. Rowe , “Unexpected Patterns of Epstein‐Barr Virus Transcription Revealed by a High Throughput PCR Array for Absolute Quantification of Viral Mrna,” Virology 474 (2015): 117–130, 10.1016/J.VIROL.2014.10.030. PMC4266535 25463610

[24] Chappell DL, White MC, Damania B. Proteomic Approaches to Investigate Gammaherpesvirus Biology and Associated Tumorigenesis,” Advances in Virus Research 109 (2021): 201–254, 10.1016/bs.aivir.2020.10.001. PMC8127003 33934828

[25] H. Guo , S. Shen , L. Wang , and H. Deng , “Role of Tegument Proteins in Herpesvirus Assembly and Egress,” Protein & Cell 1, no. 11 (2010): 987–998, 10.1007/s13238-010-0120-0. PMC4875148 21153516

[26] P. Lange and B. Damania , “Kaposi Sarcoma‐Associated Herpesvirus (KSHV),” Trends in Microbiology 28, no. 3 (2020): 236–237, 10.1016/j.tim.2019.10.006. 31759828

[27] R. Manservigi and E. Cassai , “The Glycoproteins of the Human Herpesviruses,” Comparative Immunology, Microbiology and Infectious Diseases 14, no. 2 (1991): 81–95, 10.1016/0147-9571(91)90123-u. 1657514

[28] A. N. Kirschner , J. Omerović , B. Popov , R. Longnecker , and T. S. Jardetzky , “Soluble Epstein‐Barr Virus Glycoproteins gH, gL, and gp42 Form a 1:1:1 Stable Complex That Acts Like Soluble gp42 in B‐Cell Fusion but Not in Epithelial Cell Fusion,” Journal of Virology 80, no. 19 (2006): 9444–9454, 10.1128/JVI.00572-06. PMC1617263 16973550

[29] L. M. Hutt‐Fletcher , “Epstein‐Barr Virus Entry,” Journal of Virology 81, no. 15 (2007): 7825–7832, 10.1128/JVI.00445-07. PMC1951282 17459936

[30] K. P. Michalak and W. Adamski , “A System‐Level Perspective on Epstein‐Barr Virus Persistence: The Partial Lytic Reactivation,” International Journal of Molecular Sciences 27, no. 7 (2026): 3337, 10.3390/ijms27073337. PMC13074004 41977512

[31] S. Jasinski‐Bergner , C. Stoehr , J. Bukur , et al., “Clinical Relevance of mIR‐Mediated HLA‐G Regulation and the Associated Immune Cell Infiltration in Renal Cell Carcinoma,” Oncoimmunology 4, no. 6 (2015): e1008805, 10.1080/2162402X.2015.1008805. PMC4485830 26155421

[32] L. Lyu , Q. Li , and C. Wang , “EBV Latency Programs: Molecular and Epigenetic Regulation and Its Role in Disease Pathogenesis,” Journal of Medical Virology 97, no. 7 (2025): e70501, 10.1002/jmv.70501. PMC12277944 40685972

[33] T. Inagaki , Y. Sato , J. Ito , et al., “Direct Evidence of Abortive Lytic Infection‐Mediated Establishment of Epstein‐Barr Virus Latency During B‐Cell Infection,” Frontiers in Microbiology 11 (2020): 575255, 10.3389/fmicb.2020.575255. PMC7888302 33613459

[34] M. Albanese , T. Tagawa , M. Bouvet , et al., “Epstein‐Barr Virus Micrornas Reduce Immune Surveillance by Virus‐Specific Cd8 + T Cells,” Proceedings of the National Academy of Sciences 113, no. 42 (2016): E6467–E6475, 10.1073/PNAS.1605884113. PMC5081573 27698133

[35] D. Nachmani , N. Stern‐Ginossar , R. Sarid , and O. Mandelboim , “Diverse Herpesvirus Micrornas Target the Stress‐Induced Immune Ligand MICB to Escape Recognition by Natural Killer Cells,” Cell Host & Microbe 5, no. 4 (2009): 376–385, 10.1016/J.CHOM.2009.03.003. 19380116

[36] T. S. Wong , S. Chen , M. J. Zhang , J. Y. Chan , and W. Gao , “Epstein‐Barr Virus‐Encoded Microrna BART7 Downregulates Major Histocompatibility Complex Class I Chain‐Related Peptide A and Reduces the Cytotoxicity of Natural Killer Cells to Nasopharyngeal Carcinoma,” Oncology Letters 16, no. 3 (2018): 2887–2892, 10.3892/OL.2018.9041. PMC6096257 30127876

[37] M. J. G. Hooykaas , M. van Gent , J. A. Soppe , et al., “EBV Microrna BART16 Suppresses Type I IFN Signaling,” Journal of Immunology 198, no. 10 (2017): 4062–4073, 10.4049/JIMMUNOL.1501605. 28416598

[38] N. Kitagawa , M. Goto , K. Kurozumi , et al., “Epstein‐Barr Virus‐Encoded Poly(A)(‐) RNA Supports Burkitt's Lymphoma Growth Through Interleukin‐10 Induction,” EMBO Journal 19, no. 24 (2000): 6742–6750, 10.1093/EMBOJ/19.24.6742. PMC305895 11118209

[39] J. Zuo and D. Y. Jin , “Immune Evasion by Epstein‐Barr Virus,” Current Topics in Microbiology and Immunology (2025): 1–39, 10.1007/82_2025_311. 40705087

[40] A. M. Price , J. Dai , Q. Bazot , et al., “Epstein‐Barr Virus Ensures B Cell Survival by Uniquely Modulating Apoptosis at Early and Late Times After Infection,” eLife 6 (2017): e22509, 10.7554/ELIFE.22509. PMC5425254 28425914

[41] B. Kempkes , D. Spitkovsky , P. Jansen‐Dürr , et al., “B‐Cell Proliferation and Induction of Early G1‐regulating Proteins by Epstein‐Barr Virus Mutants Conditional for Ebna2,” EMBO Journal 14, no. 1 (1995): 88–96, 10.1002/J.1460-2075.1995.TB06978.X. PMC398055 7828599

[42] D. Hanahan and R. A. Weinberg , “Hallmarks of Cancer: The Next Generation,” Cell 144, no. 5 (2011): 646–674, 10.1016/j.cell.2011.02.013. 21376230

[43] R. S. Scott , “Epstein‐Barr Virus: A Master Epigenetic Manipulator,” Current Opinion in Virology 26 (2017): 74–80, 10.1016/j.coviro.2017.07.017. PMC5742862 28780440

[44] J. Yu , Y. Wang , Q. Liu , X. Zhou , E. S. Robertson , and Y. Pei , “Genome‐Wide Mapping of EBV‐Induced Genomic Variations Identifies the Role of MUC19 in Ebv Latency,” mBio 16, no. 11 (2025): e0205525, 10.1128/mbio.02055-25. PMC12607598 40996224

[45] M. Bauer , C. M. Schöbel , C. Wickenhauser , B. Seliger , and S. Jasinski‐Bergner , “Deciphering the Role of Alternative Splicing in Neoplastic Diseases for Immune‐Oncological Therapies,” Frontiers in Immunology 15 (2024): 1386993, 10.3389/fimmu.2024.1386993. PMC11082354 38736877

[46] L. Zhang , R. Wang , and Z. Xie , “The Roles of Dna Methylation on the Promotor of the Epstein‐Barr Virus (EBV) Gene and the Genome in Patients With Ebv‐Associated Diseases,” Applied Microbiology and Biotechnology 106, no. 12 (2022): 4413–4426, 10.1007/S00253-022-12029-3. PMC9259528 35763069

[47] J. Zhao , Q. Liang , K. F. Cheung , et al., “Genome‐Wide Identification of Epstein‐Barr Virus‐Driven Promoter Methylation Profiles of Human Genes in Gastric Cancer Cells,” Cancer 119, no. 2 (2013): 304–312, 10.1002/CNCR.27724. 22833454

[48] C. L. Tsai , H. P. Li , Y. J. Lu , et al., “Activation of DNA Methyltransferase 1 by EBV LMP1 Involves c‐Jun NH(2)‐terminal Kinase Signaling,” Cancer Research 66, no. 24 (2006): 11668–11676, 10.1158/0008-5472.CAN-06-2194. 17178861

[49] C. N. Tsai , C. L. Tsai , K. P. Tse , H. Y. Chang , and Y. S. Chang , “The Epstein‐Barr Virus Oncogene Product, Latent Membrane Protein 1, Induces the Downregulation of E‐Cadherin Gene Expression via Activation of DNA Methyltransferases,” Proceedings of the National Academy of Sciences 99, no. 15 (2002): 10084–10089, 10.1073/PNAS.152059399. PMC126628 12110730

[50] H. Peng , Y. Chen , P. Gong , et al., “Higher Methylation Intensity Induced by EBV LMP1 via NF‐κB/DNMT3b Signaling Contributes to Silencing of Pten Gene,” Oncotarget 7, no. 26 (2016): 40025–40037, 10.18632/ONCOTARGET.9474. PMC5129989 27223069

[51] M. B. Dhiab , S. Ziadi , S. Mestiri , R. B. Gacem , F. Ksiaa , and M. Trimeche , “DNA Methylation Patterns in Ebv‐Positive and EBV‐Negative Hodgkin Lymphomas,” Cellular oncology (Dordrecht, Netherlands) 38, no. 6 (2015): 453–462, 10.1007/S13402-015-0242-8. PMC13004184 26350502

[52] L. Cai , Y. Ye , Q. Jiang , et al., “Retracted Article: Epstein–Barr Virus‐Encoded Microrna BART1 Induces Tumourmetastasis by Regulating Pten‐Dependent Pathways in Nasopharyngealcarcinoma,” Nature Communications 6 (2015): 7353, 10.1038/NCOMMS8353. PMC4507016 26135619

[53] Y. J. Xu , R. Zhou , J. F. Zong , et al., “Epstein‐Barr Virus‐Coded miR‐BART13 Promotes Nasopharyngeal Carcinoma Cell Growth and Metastasis via Targeting of the NKIRAS2/NF‐κB Pathway,” Cancer Letters 447 (2019): 33–40, 10.1016/J.CANLET.2019.01.022. 30684592

[54] M. Samanta , D. Iwakiri , T. Kanda , T. Imaizumi , and K. Takada , “EB Virus‐Encoded Rnas Are Recognized by RIG‐I and Activate Signaling to Induce Type I IFN,” EMBO Journal 25, no. 18 (2006): 4207–4214, 10.1038/SJ.EMBOJ.7601314. PMC1570431 16946700

[55] T. Hohmann , U. Hohmann , F. Dehghani , O. Grisk , and S. Jasinski‐Bergner , “Identification and Characterization of the RNA Modifying Factors PUS7 and WTAP as Key Components for the Control of Tumor Biological Processes in Renal Cell Carcinomas,” Current Issues in Molecular Biology 47, no. 4 (2025): 266, 10.3390/cimb47040266. PMC12025953 40699665

[56] S. Jasinski‐Bergner , J. Blümke , C. Wickenhauser , and B. Seliger , “Relevance of 2’‐O‐Methylation and Pseudouridylation for the Malignant Melanoma,” Cancers 13, no. 5 (2021): 1167, 10.3390/cancers13051167. PMC7963185 33803145

[57] X. Zheng , J. Wang , X. Zhang , et al., “RNA m6 A Methylation Regulates Virus‐Host Interaction and EBNA2 Expression During Epstein‐Barr Virus Infection,” Immunity, Inflammation and Disease 9, no. 2 (2021): 351–362, 10.1002/IID3.396. PMC8127537 33434416

[58] Y. Yanagi , T. Watanabe , Y. Hara , Y. Sato , H. Kimura , and T. Murata , “EBV Exploits RNA m6A Modification to Promote Cell Survival and Progeny Virus Production During Lytic Cycle,” Frontiers in Microbiology 13 (2022): 870816, 10.3389/FMICB.2022.870816. PMC9240777 35783391

[59] M. E. Holmes and K. J. Hertel , “Interdependent Regulation of Alternative Splicing by SR and hnRNP Proteins,” *bioRxiv* (2024), 10.1101/2024.08.19.608666.

[60] A. Piton , “NOVA1/2 Genes and Alternative Splicing in Neurodevelopment,” Current Opinion in Genetics & Development 93 (2025): 102373, 10.1016/J.GDE.2025.102373. 40555137

[61] P. F. Chen , J. S. Hsiao , C. L. Sirois , and S. J. Chamberlain , “RBFOX1 and RBFOX2 Are Dispensable in iPSCs and Ipsc‐Derived Neurons and Do Not Contribute to Neural‐Specific Paternal UBE3A Silencing,” Scientific Reports 6 (2016): 25368, 10.1038/SREP25368. PMC4857170 27146458

[62] E. T. Wang , A. J. Ward , J. M. Cherone , et al., “Antagonistic Regulation of MRNA Expression and Splicing by CELF and MBNL Proteins,” Genome Research 25, no. 6 (2015): 858–871, 10.1101/GR.184390.114. PMC4448682 25883322

[63] M. Romanelli , E. Diani , and P. Lievens , “New Insights into Functional Roles of the Polypyrimidine Tract‐Binding Protein,” International Journal of Molecular Sciences 14, no. 11 (2013): 22906–22932, 10.3390/IJMS141122906. PMC3856098 24264039

[64] G. Pimienta , V. Fok , M. Haslip , M. Nagy , S. Takyar , and J. A. Steitz , “Proteomics and Transcriptomics of BJAB Cells Expressing the Epstein‐Barr Virus Noncoding RNAs EBER1 and EBER2,” PLoS One 10, no. 6 (2015): e0124638, 10.1371/journal.pone.0124638. PMC4487896 26121143

[65] N. Lee , G. Pimienta , and J. A. Steitz , “AUF1/hnRNP D Is a Novel Protein Partner of the EBER1 Noncoding RNA of Epstein‐Barr Virus,” RNA 18, no. 11 (2012): 2073–2082, 10.1261/rna.034900.112. PMC3479396 23012480

[66] N. Lee , T. A. Yario , J. S. Gao , and J. A. Steitz , “Ebv Noncoding RNA EBER2 Interacts With Host RNA‐Binding Proteins to Regulate Viral Gene Expression,” Proceedings of the National Academy of Sciences 113, no. 12 (2016): 3221–3226, 10.1073/pnas.1601773113. PMC4812724 26951683

[67] D. Verma and S. Swaminathan , “Epstein‐Barr Virus SM Protein Functions as an Alternative Splicing Factor,” Journal of Virology 82, no. 14 (2008): 7180–7188, 10.1128/JVI.00344-08. PMC2446978 18463151

[68] H. H. Chua , T. Kameyama , A. Mayeda , and T. H. Yeh , “Epstein‐Barr Virus Enhances Cancer‐Specific Aberrant Splicing of TSG101 Pre‐mRNA,” International Journal of Molecular Sciences 23, no. 5 (2022): 2516, 10.3390/IJMS23052516. PMC8910672 35269659

[69] S. Boudreault , P. Roy , G. Lemay , and M. Bisaillon , “Viral Modulation of Cellular RNA Alternative Splicing: A New Key Player in Virus‐Host Interactions?,” WIREs RNA 10, no. 5 (2019): e1543, 10.1002/wrna.1543. PMC6767064 31034770

[70] R. Park and G. Miller , “Epstein‐Barr Virus‐Induced Nodules on Viral Replication Compartments Contain RNA Processing Proteins and a Viral Long Noncoding RNA,” Journal of Virology 92, no. 20 (2018): e01254–18, 10.1128/JVI.01254-18. PMC6158406 30068640

[71] V. E. S. Armero , M. P. Tremblay , A. Allaire , et al., “Transcriptome‐Wide Analysis of Alternative RNA Splicing Events in Epstein‐Barr Virus‐Associated Gastric Carcinomas,” PLoS One 12, no. 5 (2017): e0176880, 10.1371/journal.pone.0176880. PMC5426614 28493890

[72] N. J. Homa , R. Salinas , E. Forte , T. J. Robinson , M. A. Garcia‐Blanco , and M. A. Luftig , “Epstein‐Barr Virus Induces Global Changes in Cellular mRNA Isoform Usage That Are Important for the Maintenance of Latency,” Journal of Virology 87, no. 22 (2013): 12291–12301, 10.1128/JVI.02464-13. PMC3807935 24027308

[73] T. Tsurumi , M. Fujita , and A. Kudoh , “Latent and Lytic Epstein‐Barr Virus Replication Strategies,” Reviews in Medical Virology 15, no. 1 (2005): 3–15, 10.1002/rmv.441. 15386591

[74] R. Küppers , “B Cells under Influence: Transformation of B Cells by Epstein‐Barr Virus,” Nature Reviews Immunology 3, no. 10 (2003): 801–812, 10.1038/nri1201. 14523386

[75] E. Manet , H. Polvèche , F. Mure , et al., “Modulation of Alternative Splicing During Early Infection of Human Primary B Lymphocytes With Epstein‐Barr Virus (EBV): A Novel Function for the Viral Ebna‐Lp Protein,” Nucleic Acids Research 49, no. 18 (2021): 10657–10676, 10.1093/NAR/GKAB787. PMC8501971 34530456

[76] R. L. Hutcheson , A. Chakravorty , and B. Sugden , “Burkitt Lymphomas Evolve to Escape Dependencies on Epstein‐Barr Virus,” Frontiers in Cellular and Infection Microbiology 10 (2020): 606412, 10.3389/fcimb.2020.606412. PMC7829347 33505922

[77] O. L. Hatton , A. Harris‐Arnold , S. Schaffert , S. M. Krams , and O. M. Martinez , “The Interplay Between Epstein‐Barr Virus and B Lymphocytes: Implications for Infection, Immunity, and Disease,” Immunologic Research 58, no. 2–3 (2014): 268–276, 10.1007/s12026-014-8496-1. PMC4199828 24619311

[78] C. Wang , X. Liu , J. Liang , et al., “A Dna Tumor Virus Globally Reprograms Host 3D Genome Architecture to Achieve Immortal Growth,” Nature Communications 14, no. 1 (2023): 1598, 10.1038/s41467-023-37347-6. PMC10033825 36949074

[79] C. Kaiser , G. Laux , D. Eick , N. Jochner , G. W. Bornkamm , and B. Kempkes , “The Proto‐Oncogene C‐Myc Is a Direct Target Gene of Epstein‐Barr Virus Nuclear Antigen 2,” Journal of Virology 73, no. 5 (1999): 4481–4484, 10.1128/JVI.73.5.4481-4484.1999. PMC104340 10196351

[80] W. W. Sung , Y. C. Chu , P. R. Chen , M. H. Liao , and J. W. Lee , “Positive Regulation of HIF‐1A Expression by EBV Oncoprotein LMP1 in Nasopharyngeal Carcinoma Cells,” Cancer Letters 382, no. 1 (2016): 21–31, 10.1016/j.canlet.2016.08.021. 27567526

[81] L. Zuo , H. Yu , L. Liu , et al., “The Copy Number of Epstein‐Barr Virus Latent Genome Correlates With the Oncogenicity by the Activation Level of LMP1 and NF‐kB,” Oncotarget 6, no. 38 (2015): 41033–41044, 10.18632/oncotarget.5708. PMC4747387 26517512

[82] L. Zhou , J. Hao , Y. Yuan , et al., “EIYMNVPV Motif Is Essential for A1CF Nucleus Localization and A1CF (‐8aa) Promotes Proliferation of MDA‐MB‐231 Cells via Up‐Regulation of Il‐6,” International Journal of Molecular Sciences 17, no. 6 (2016): 811, 10.3390/IJMS17060811. PMC4926345 27231908

[83] X. Yan , Q. Li , D. Ni , et al., “Apobec‐1 Complementation Factor Regulates Cell Migration and Apoptosis Through Dickkopf1 by Acting on Its 3′ Untranslated Region in MCF7 Cells,” Tumor Biology 39, no. 6 (2017): 101042831770621, 10.1177/1010428317706218. 28639893

[84] Y. Huang , L. Xiao , M. Y. Daba , et al., “Characterization of Molecular Subtypes Based on Chromatin Regulators and Identification of the Role of NPAS2 in Lung Adenocarcinoma,” Clinical Epigenetics 15, no. 1 (2023): 72, 10.1186/S13148-023-01486-W. PMC10149025 37120564

[85] R. Lee , K. Hirano , S. Anant , D. Baunoch , and N. Davidson , “An Alternatively Spliced Form of apobec‐1 Messenger Rna Is Overexpressed in Human Colon Cancer,” Gastroenterology 115, no. 5 (1998): 1096–1103, 10.1016/S0016-5085(98)70080-0. 9797364

[86] D. Xu , A. N. Forbes , S. Cohen , A. Palladino , T. Karadimitriou , and E. Khurana , “Recapitulation of Patient‐Specific 3D Chromatin Conformation Using Machine Learning,” Cell Reports Methods 3, no. 9 (2023): 100578, 10.1016/J.CRMETH.2023.100578. PMC10545938 37673071

[87] Q. Liu , C. Y. Chen , and G. L. Chen , “High APOBEC1 Complementation Factor Expression Positively Modulates the Proliferation, Invasion, and Migration of Endometrial Cancer Cells Through Regulating P53/P21 Signaling Pathway,” Cancer Biotherapy & Radiopharmaceuticals 37, no. 9 (2022): 750–758, 10.1089/CBR.2020.3957. 32818382

[88] D. Ni , Q. Yi , J. Liu , et al., “A1CF‐promoted Colony Formation and Proliferation of Rcc Depends on DKK1‐MEK/ERK Signal Axis,” Gene 730 (2020): 144299, 10.1016/J.GENE.2019.144299. 31881249

[89] H. Xia , Y. Liu , L. Xu , H. Mao , Q. Zhou , and Y. Xie , “APOBEC1 Complementation Factor Facilitates Cell Migration by Promoting Nucleus Translocation of SMAD3 in Renal Cell Carcinoma Cells,” In Vitro Cellular & Developmental Biology ‐ Animal 57, no. 5 (2021): 501–509, 10.1007/S11626-021-00589-Z. 34002283

[90] L. Wang and Q. Cheng , “APOBEC‐1 Complementation Factor: From RNA Binding to Cancer,” Cancer Control 31 (2024), 10.1177/10732748241284952. PMC11439182 39334524

[91] L. Hong , P. Yang , L. Zhang , et al., “The VAX2‐LINC01189‐hnRNPF Signaling Axis Regulates Cell Invasion and Migration in Gastric Cancer,” Cell Death Discovery 9, no. 1 (2023): 387, 10.1038/S41420-023-01688-4. PMC10590441 37865686

[92] S. Ma , Z. Wang , Z. Xiong , et al., “Enhancer Transcription Profiling Reveals an Enhancer Rna‐Driven Ferroptosis and New Therapeutic Opportunities in Prostate Cancer,” Signal Transduction and Targeted Therapy 10, no. 1 (2025): 87, 10.1038/S41392-025-02170-6. PMC11906896 40082405

[93] Y. Jiang , W. Liao , Q. Xin , et al., “Nuclear and Cytoplasmic USP30‐AS1 Coordinately Regulate Breast Cancer Progression Through HnRNPF/p21 and EZH2/c‐Myc/p21 Axes,” Genes & Diseases 13, no. 2 (2025): 101684, 10.1016/J.GENDIS.2025.101684. PMC12765101 41492473

[94] H. Mikami , S. Noguchi , J. Akatsuka , et al., “snRNAs From Radical Prostatectomy Specimens Have the Potential to Serve as Prognostic Factors for Clinical Recurrence After Biochemical Recurrence in Patients With High‐Risk Prostate Cancer,” Cancers 16, no. 9 (2024): 1757, 10.3390/CANCERS16091757. PMC11083327 38730709

[95] U. Korsgaard , J. L. García‐Rodríguez , T. Jakobsen , et al., “The Transcriptional Landscape of Coding and Noncoding RNAs in Recurrent and Nonrecurrent Colon Cancer,” American Journal of Pathology 194, no. 8 (2024): 1424–1442, 10.1016/J.AJPATH.2024.04.003. 38704091

[96] B. Wang , C. Yin , X. Yang , et al., “Six Genes Associated With Lymphatic Metastasis in Colon Adenocarcinoma Linked to Prognostic Value and Tumor Immune Cell Infiltration,” Evidence‐Based Complementary and Alternative Medicine: eCAM 2022 (2022): 4304361, 10.1155/2022/4304361. PMC9444393 36072412

[97] A. Augspach , K. D. Drake , L. Roma , et al., “Minor Intron Splicing Is Critical for Survival of Lethal Prostate Cancer,” Molecular Cell 83, no. 12 (2023): 1983–2002.e11, 10.1016/J.MOLCEL.2023.05.017. PMC10637423 37295433

[98] M. T. Moreno‐Montilla , S. Pedraza‐Arevalo , A. Martínez‐López , et al., “Exploring RNA Biology in Pseudomyxoma Peritonei Uncovers Splicing Dysregulation as a Novel, Targetable Molecular Vulnerability,” Cancer Gene Therapy 32, no. 6 (2025): 721–736, 10.1038/S41417-025-00911-X. PMC12183082 40301643

[99] M. C. Vázquez‐Borrego , A. C. Fuentes‐Fayos , E. Venegas‐Moreno , et al., “Splicing Machinery Is Dysregulated in Pituitary Neuroendocrine Tumors and Is Associated With Aggressiveness Features,” Cancers 11, no. 10 (2019): 1439, 10.3390/CANCERS11101439. PMC6826715 31561558

[100] A. G. K. Roberts , D. R. Catchpoole , and P. J. Kennedy , “Identification of Differentially Distributed Gene Expression and Distinct Sets of Cancer‐Related Genes Identified by Changes in Mean and Variability,” NAR Genomics and Bioinformatics 4, no. 1 (2022): 124, 10.1093/NARGAB/LQAB124. PMC8759562 35047816

[101] S. Shuai , H. Suzuki , A. Diaz‐Navarro , et al., “The U1 Spliceosomal Rna Is Recurrently Mutated in Multiple Cancers,” Nature 574, no. 7780 (2019): 712–716, 10.1038/s41586-019-1651-z. 31597163

[102] H. Guo , J. Zuo , G. Hu , et al., “Silencing of Small Nuclear Ribonucleoprotein Polypeptide B Inhibits the Progression of Esophageal Squamous Cell Carcinoma,” Oncology Letters 31, no. 5 (2026): 1–10, 10.3892/OL.2026.15561. PMC13082865 41993852

[103] X. Wang , H. Zhang , Z. Guo , et al., “SNRPB Promotes the Progression of Hepatocellular Carcinoma via Regulating Cell Cycle, Oxidative Stress, and Ferroptosis,” Aging 16, no. 1 (2024): 348–366, 10.18632/AGING.205371. PMC10817389 38189879

[104] M. Shimojo , Y. Kasahara , M. Inoue , et al., “A Gapmer Antisense Oligonucleotide Targeting SRRM4 Is a Novel Therapeutic Medicine for Lung Cancer,” Scientific Reports 9, no. 1 (2019): 7618, 10.1038/S41598-019-43100-1. PMC6527545 31110284

[105] K. Deshpande , V. Martirosian , B. N. Nakamura , et al., “SRRM4‐mediated REST to REST4 Dysregulation Promotes Tumor Growth and Neural Adaptation in Breast Cancer Leading to Brain Metastasis,” Neuro‐Oncology 26, no. 2 (2024): 309–322, 10.1093/NEUONC/NOAD175. PMC10836770 37716001

[106] G. Hu , H. Yao , Z. Wei , et al., “A Bioinformatics Approach to Identify a Disulfidptosis‐Related Gene Signature for Prognostic Implication in Colon Adenocarcinoma,” Scientific Reports 13, no. 1 (2023): 12403, 10.1038/S41598-023-39563-Y. PMC10390519 37524774

[107] M. T. Wang , C. H. Ni , Y. Q. Lu , et al., “Next‐Generation Sequencing in Early‐Stage Multiple Primary Lung Cancer: The Prognostic Significance of Genomic Accumulation Status and BCL2l11del,” Translational Oncology 55 (2025): 102383, 10.1016/J.TRANON.2025.102383. PMC12002891 40186984

[108] S. J. Hernandez , D. M. Dolivo , and T. Dominko , “PRMT8 Demonstrates Variant‐Specific Expression in Cancer Cells and Correlates With Patient Survival in Breast, Ovarian and Gastric Cancer,” Oncology Letters 13, no. 3 (2017): 1983–1989, 10.3892/OL.2017.5671. PMC5403539 28454353

[109] J. Rius , M. Guma , C. Schachtrup , et al., “NF‐κB Links Innate Immunity to the Hypoxic Response Through Transcriptional Regulation of HIF‐1Α,” Nature 453, no. 7196 (2008): 807–811, 10.1038/nature06905. PMC2669289 18432192

[110] A. Colaprico , T. C. Silva , C. Olsen , et al., “TCGAbiolinks: An R/Bioconductor Package for Integrative Analysis of TCGA Data,” Nucleic Acids Research 44, no. 8 (2016): e71, 10.1093/nar/gkv1507. PMC4856967 26704973

[111] M. I. Love , W. Huber , and S. Anders , “Moderated Estimation of Fold Change and Dispersion for RNA‐Seq Data With DESeq2,” Genome Biology 15, no. 12 (2014): 550, 10.1186/s13059-014-0550-8. PMC4302049 25516281

[112] Z. Gu , R. Eils , and M. Schlesner , “Complex Heatmaps Reveal Patterns and Correlations in Multidimensional Genomic Data,” Bioinformatics 32, no. 18 (2016): 2847–2849, 10.1093/BIOINFORMATICS/BTW313. 27207943

[113] S. X. Ge , D. Jung , and R. Yao , “Shinygo: A Graphical Gene‐Set Enrichment Tool for Animals and Plants,” Bioinformatics 36, no. 8 (2020): 2628–2629, 10.1093/BIOINFORMATICS/BTZ931. PMC7178415 31882993

